# The rK39 Antigen from an Iranian Strain of *Leishmania infantum*: Detection of Anti-*Leishmania* Antibodies in Humans and Dogs

**Published:** 2020

**Authors:** Bibi Razieh HOSSEINI FARASH, Mehdi MOHEBALI, Bahram KAZEMI, Homa HAJJARAN, Abdolmajid FATA, Reza RAOOFIAN, Behnaz AKHOUNDI, Majid MOJARRAD, Pietro MASTROENI, Mohammad Kazem SHARIFI-YAZDI, Mohammad Hossein TANIPOUR

**Affiliations:** 1. Department of Medical Parasitology and Mycology, School of Medicine, Mashhad University of Medical Sciences, Mashhad, Iran; 2. Department of Medical Parasitology and Mycology, School of Public Health, Tehran University of Medical Sciences, Tehran, Iran; 3. Cutaneous Leishmaniasis Research Center, Mashhad University of Medical Sciences, Mashhad, Iran; 4. Center for Research of Endemic Parasites of Iran, Tehran University of Medical Sciences, Tehran, Iran; 5. Department of Biotechnology, School of Medicine, Shahid Beheshti University of Medical Sciences, Tehran, Iran; 6. Legal Medicine Research Center, Legal Medicine Organization, Tehran, Iran; 7. Department of Medical Genetics, School of Medicine, Mashhad University of Medical Sciences, Mashhad, Iran; 8. Department of Veterinary Medicine, University of Cambridge, Cambridge, United Kingdom; 9. Center for Research of Zoonoses of Iran, Tehran University of Medical Sciences, Tehran, Iran

**Keywords:** rK39 recombinant antigen, *Leishmania infantum*, Visceral leishmaniasis, Human, Dog

## Abstract

**Background:**

Visceral leishmaniasis (VL) is the most severe form of leishmaniasis in Iran with high mortality rates in the case of inaccurate diagnosis and treatment. This study aimed to prepare and evaluate a new rk39 recombinant antigen from an Iranian strain of *Leishmania infantum* for diagnosis of VL in humans and dogs.

**Methods:**

rK39-based enzyme-linked immunosorbent assay (ELISA) was compared with the direct agglutination test (DAT) for the detection of anti *L. infantum* antibodies. We screened 84 human sera and 87 dog sera from clinical cases in the endemic area of Meshkin-Shahr, Iran along with 176 sera from healthy controls (collected from 86 humans and 90 dogs) during 2013–2016.

**Results:**

Using the rK39 ELISA, a sensitivity of 85.7% (95% CI, 95–99%) and a specificity of 86.0% (95% CI, 95%–99%) were detected in human sera at a 1:800 (cut-off) titer when DAT-confirmed cases were compared with healthy controls; a sensitivity of 96.6% (95% CI, 95%–99%) and specificity of 94.4% (95% CI, 95%–99%) were found at a 1:80 (cut-off) titer compared with DAT. Kappa analysis indicated agreement between the rK39 ELISA and DAT (0.718) when using human sera at a 1:800 (cut-off) titer as well as (0.910) at a 1:80 (cut-off) titer when using dog sera (*P*<0.05).

**Conclusion:**

New rk39 recombinant antigen from an Iranian strain of *Leishmania infantum* seems to be used for diagnosis of VL in humans and dogs. Further extended field studies are recommended.

## Introduction

Visceral leishmaniasis (VL) is a parasitic disease with high levels of morbidity and mortality. Dogs are the main reservoir host for the Mediterranean type of VL, which is endemic in some areas of Iran (1,2).

Visualization of the parasite in bone marrow, liver or splenic aspirates and isolation by culture are currently available diagnostic procedures, but these are invasive or not sensitive enough for the diagnosis all forms of VL (3).

rK39, which includes a 39-aminoacid-repetitive immune-dominant epitope of the kinesin-related antigen, has shown high sensitivity (93% to 100%), specificity (97% to 98%) and strong antigenicity even among HIV co-infected patients (sensitivity 81%) compared to other recombinant VL antigens(1, 3–5).

The serological assay based on crude antigen (C-Ag) preparation has also been commonly used to diagnose this *Leishmania* disease; however, the C-Ag assay has shown limitations in terms of specificity, sensitivity and has also shown cross-reactivity with sera from patients with trypanosomiasis, tuberculosis and toxoplasmosis (3).

Dipstick tests produced in the USA and Europe using rK39 from *L. infantum* (rK39 RDT) have been introduced by Iranian researchers as a rapid and non-invasive method to identify VL, especially in suspected symptomatic reservoirs(6,7).

Due to difficulties in cloning the repetitive and GC rich sequence of the rk39 gene, only one study has so far been performed using the rK39-sub recombinant Ag from an Iranian strain of *L. infantum*. Therefore, the attempts to optimize appropriate antigens for the rapid diagnosis of VL in humans and dogs have not been successful in Iran (2,5,8). The Direct Agglutination Test (DAT) is a serological test with good reproducibility, sensitivity and specificity in Iranian settings (9,10). However, the need for prolonged incubation times (12 to 18 h) and for serial serum dilutions are limitations of DAT (7,11).

The main goal of this study was to evaluate rk39 recombinant antigen from an Iranian strain of *L. infantum* to develop a local diagnostic test and to determine the suitability of this antigen in enzyme-linked immunosorbent assay (ELISA).

## Methods

### Ethical consideration

This study was approved by the Ethical Committee of Tehran University of Medical Sciences (Ethical code no. 92-03-162-24558) in accordance with the Helsinki Declaration and guidelines. Dog sera were collected in coordination with Iran Veterinary Organization after informed consent from the owners.

The human sera were collected from volunteers following informed consent. Children were included in this study after consent from their legal guardians.

### Parasite and Culture

The *L. infantum* strain (MCAN/IR/14/M14 with GenBank Accession number KT201383) used in this project was isolated from a VL infected dog in Meshkin-Shahr. Promastigote culture and DNA extraction were performed (12).

### PCR amplification of L. infantum k39 gene

To amplify the K39 gene, primers were designed based on *L. chagasi* sequence (GenBank Accession number AF131228), forward, F, 5′- GGATCCATGGCAGCCGAACTTGATGCCG -3′ containing a BamHI site and reverse, R, 5′- CTCGAGCTGGCTCGCCAGCTCCG -3′ which contained a *XhoI* site for k39 gene, and amplified by ABI DNA thermal cycler. The mixture for the PCR reaction included 1.5 μl (5pm) of each primer, 0.5 μl dNTPs, 0.5 μl Mgcl2 and 0.5 μl DMSO. Amplification was performed at 94 °C for 5 min, followed by 35 cycles at 94 °C for 1 min, 68 °C for 1 min, 72 °C for 1 min and 30 sec and a final extension for 20 min.

### Cloning of PCR products and confirmation of pCR®-TOPO® -k39 and pET-32(+)-k39 Cloning

The k39 band, of a predicted length of approximately 843 bp, was ligated into pCR®-TOPO® vector by TOPO®TA Cloning® Kit (Invitrogen, USA) and transformed into *E. coli* (DH5-alpha competent cells). Plasmid DNA was extracted using the YTA Miniprep Kit (Yekta Tajhiz Azma Co, Tehran, Iran) and digested using BamH1 and *XhoI.* The insert was subcloned into pET-32a (+).

PCR screening in the pCR®-TOPO® and pET-32a (+) recombinant plasmids was performed by M13 and T7 terminator primers. The recombinant plasmids were digested using BamHI and XhoI to confirm insertion of the rK39 gene.

### Expression, purification and confirmation of L. infantum rK39 protein

The recombinant pET-32a (+) with N and C -terminal His-tag fusions for affinity purification was expressed, purified and confirmed (2,12).

### Preparation of promastigote crude antigen for ELISA

Stationary-phase promastigotes of *L. infantum* were washed in cold PBS for 3 times and ruptured by sonication. The crude Ag in the supernatant was stored at −20 °C after centrifugation at 4500 rpm for 20 min at 4 °C. Protein concentration was estimated by Lowry’s method (13).

### Study population

The sample size was determined based on the sensitivity and specificity of 70% obtained from studies done on rK39 dipstick test for diagnosis the sera infected by VL in Iran(14).

Serum samples were collected between Apr 2013 and Mar 2016, using a non-probability convenience sampling technique from 171 confirmed VL- infected sera introduced by Iranian Reference Laboratory for Leishmaniasis in Tehran.

All the cases showed anti-*Leishmania* antibodies using DAT (≥1:800 for human and ≥1:80 for dog) including 84 humans and 87 dogs from the endemic area of Meshkin-Shahr, Iran.

Most of the canine sera were obtained from symptomatic dogs with cutaneous lesions, alopecia, diarrhea and titers of anti-*Leishmania* antibodies ≥1:320.

In parallel, 176 healthy control samples were obtained from geographical areas not endemic for VL (86 human and 90 dog samples) along with 10 patient sera and 8 canine sera with unrelated infectious diseases (malaria, tuberculosis, cutaneous leishmaniasis, toxoplasmosis and hydatidosis in human, toxocariasis, toxoplasmosis, cutaneous leishmaniasis and babesiosis in dog).

### ELISA

A checkerboard titration was carried out to optimize the concentration of rK39 and CA using standard ELISA methods.

ELISA plates (Nunc™ MaxiSorp™) were coated overnight with rK39 (1 μg/ml) and crude antigen (6 μg/ml) at 4 °C in carbonate/bicarbonate buffer at pH=9.6. All the samples were analyzed by ELISA (2).

The optical density (OD) was measured at 492 nm and entered into MedCalc (ver. 17.7.2). The ROC- curve was created for each Ag separately in this software.

### Direct Agglutination Test (DAT)

DAT antigen was obtained by the *Leishmania* laboratory of the school of public health at Tehran University of Medical Sciences. All the samples were tested according to standard methods (2)*.*

Based on previous studies on the diagnosis of asymptomatic stages of VL, titers equal or higher than 1:800 and 1:80 were considered as the cut-off point for human and dog sera, respectively (15–17)*.* We considered those individuals who had a level of antibody titers less than 1:3200 using DAT and without special clinical signs related to VL as being in the asymptomatic stage of the infection. The individuals who developed antibodies (equal or higher than 1:3200) and signs of VL were considered as being in the *symptomatic stage*.

### Statistical analysis

The sensitivity and specificity, positive predictive and negative predictive values were calculated in comparison with DAT. Moreover, Kappa (*k*) values with 95% confidence intervals were determined to show the degree of agreement between expected result (rK39 ELISA, CA-ELISA results) and actual result (DAT results) based on criteria scales (18). The correlation between different assays with DAT was carried out using McNamara’s test and analyzed by MedCalc software (ver. *17.7.2)*. In order to determine the diagnostic efficacy.

## Results

### Amplification and cloning of L. infantum k39 gene

Amplification of K39 of *L. infantum* (MCAN/IR/14/M14 with GenBank Accession number KT201383) showed 843 bp band of interest.

### Confirmation of pCR®-TOPO® -k39 and pET-32(+)-k39 Cloning

**Fig. 1:**
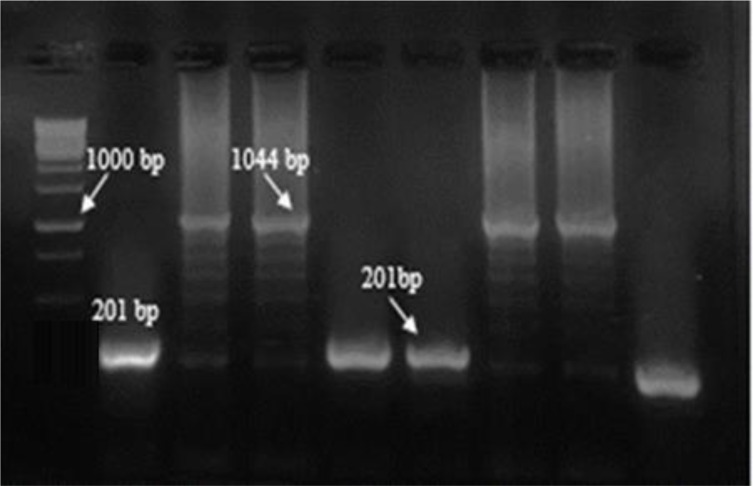
PCR results of screening of cloned plasmids after ligation into pCR®-TOPO® with M13 primers Left to right: M, molecular-weight standard 1kbp; lanes 3, 4,7,8 cloned plasmids for k39, respectively; lanes 2, 5,6 non-cloned plasmids; lane 9, Negative control

**Fig. 2:**
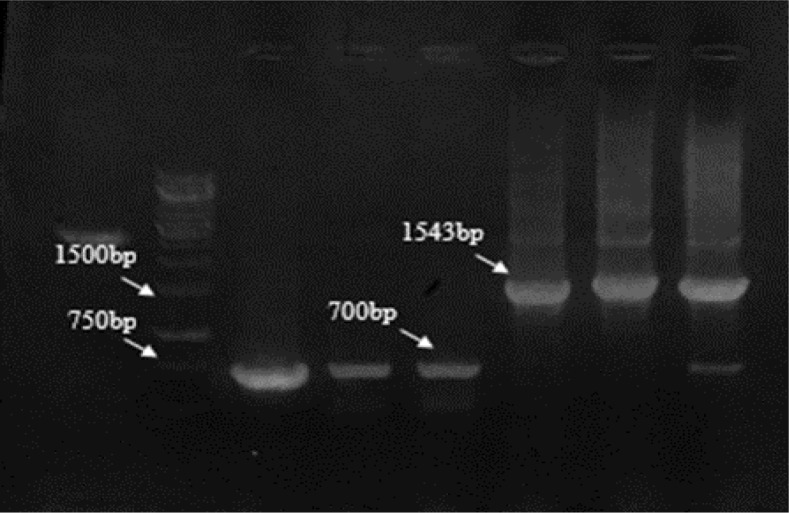
PCR results of screening of cloned plasmids after ligation into pET-32a (+) with T7 terminator primers Left to right: M, molecular-weight standard 1kbp; lanes 5, 6, 7 cloned plasmids for k39, respectively; lanes 3,4 non-cloned plasmids; lane 2, Negative control

PCR screening in pCR®-TOPO® plasmids was carried out by F&R M13 primers. The recombinant bands were 1044 and the nonrecombinant ones yielded a product of 201bp (Fig. 1) while the recombinant pET-32a (+) plasmids showed a 1543 bp band in comparison the non-recombinants about 700bp by F&R T7 terminator primers (Fig. 2).

The both of plasmids were digested by restriction enzymes (BamHI and XhoI) and revealed two different bands.

### Expression, purification and confirmation of L. infantum rK39 protein

The *L. infantum* k39 gene with N and C -terminal His-tag fusions were expressed in the pET 32a (+) vector. The recombinant protein was purified from the soluble fraction by NIIDA resin affinity chromatography. Western blotting using pooled serum of 20 VL-confirmed patients and anti-His antibodies revealed a single band of 58 kD. The yield of protein ranged from 47.8 to 55 μgr/ml after purification and dialysis.

### Evaluation of L. infantum rK39 antigen for visceral leishmaniasis with sera of VL patients

The sensitivity and specificity of the *L. infantum* rK39 antigen were determined by ELISA compared to DAT and CA- ELISA (Tables 1 and 2).

**Table 1:** Comparison of rK39- ELISA and DAT for diagnosis of human *L. infantum* infection

***DAT***			***rK39- ELISA***					
	***Positive***		***Negative***		***Total***	
	***No.***	***%***	***No.***	***%***	***No.***	***%***
1:800	3	4.2	7	58.3	10	11.9
1:1600	17	23.7	5	41.7	22	26.2
1:3200	15	20.8	0	0	15	17.9
1:6400	8	11.1	0	0	8	9.6
1:12800	10	13.9	0	0	10	11.9
1:51200	6	8.3	0	0	6	7.1
1:102400	13	18	0	0	13	15.4
Total	72	100	12	100	84	100

**Table 2:** Comparison of rK39- ELISA and DAT for diagnosis of canine *L. infantum* infection

***DAT***			***rK39- ELISA***					
	***Positive***		***Negative***		***Total***	
	***No.***	***%***	***No.***	***%***	***No.***	***%***
1:160	2	2.4	1	33.33	3	3.5
1:320	2	2.4	1	33.33	3	3.5
1:640	13	15.5	0	0	13	14.9
1:1280	15	17.9	0	0	15	17.2
1:2560	4	4.7	0	0	4	4.6
1:5120	19	22.6	0	0	19	21.8
1:20480	29	34.5	1	33.33	30	34.5
Total	84	100	3	100	87	100

Data analysis by the SPSS and MedCalc software showed that the sensitivity and specificity of the rK39- ELISA were 85.7% and 86% in humans and 96.6% and 94.4% in dogs, respectively while these values were less for CA-ELISA with 75% and 52.3% in humans, 93.1% and 83.3% in dogs, respectively (Table 3). The sensitivity of rK39- ELISA for sera from symptomatic (≥3200) (n= 52) and asymptomatic (≤1600) (n= 32) individuals were 100% and 62.5%, respectively with a similar level of specificity. A sensitivity of 85.2% was achieved for symptomatic humans using CA-ELISA, which also showed an inferior sensitivity of about 59.4% for sera from asymptomatic individuals. The specificity of the CA-ELISA in people with high titers in DAT (≥3200) was 53.5%, while this value was 52.3% in people having lower DAT titers (≤1600).

**Table 3:** Sensitivity, specificity, positive predictive value, and negative predictive value of Crude Ag- ELISA and rK39 ELISA tests for serodiagnosis of infected VL samples

***Reservoir***	***Tests***	***Sn (%)***	***Sp (%)***	***PPV (%)***	***NPV (%)***
Human	rK39-ELISA	85.7	86	86.7	93.7
	Crude Ag- ELISA	75	53.5	61.2	68.7
Dog	rK39-ELISA	96.6	94.4	94.4	96.6
	Crude Ag- ELISA	93.3	83.3	84.6	92.6

Sn, Sensitivity; Sp, Specificity; PPV, Positive predictive value; NPV, Negative predictive value.

There were no significant differences among results obtained from rK39-ELISA and DAT in humans *P*=0.143 and dogs *P*=0.727 by McNamara’s test. There was a significant difference in the comparison of the CA-ELISA with DAT for human samples (*P*=0.02), but this was not observed for dogs (*P*=0.078).

The level of agreement of rK39-ELISA and CA-ELISA with DAT was measured by *k* index using the SPSS software. The data indicated a moderate agreement between rK39-ELISA and DAT (*k* =0.485), but no concordance of CA-ELISA with DAT in asymptomatic humans (*k* =0.092); conversely the degree of agreement for symptomatic humans was very high using the rK39-ELISA (*k* =0.823), but weak in CA-ELISA (*k* =0.340). Totally, *k* coefficient for rK39-ELISA and CA-ELISA were 0.711 and 0.284, respectively in humans. These agreement values showed almost perfect agreement in dogs for rK39 and a significant result for CA with DAT (*k*=0.910 for rK39- ELISA and *k* =0.763 for CA-ELISA).

The Youden’s indexes for rK39-ELISA and CA-ELISA were 0.720, and 0.285 in human and 0.910 and 0.764 in dog reservoirs, respectively that indicate a high accuracy for rK39-ELISA in human and canine while an acceptable accuracy was seen for CA-ELISA in dog.

## Discussion

The present study reports for the first time the successful cloning and expression of the recombinant k39 antigen from an Iranian strain of *L. infantum*. The cloning of this antigen could be challenging due to high GC content in the gene (more than 67%), secondary structures and repetitive sequences. Repetitive DNA made problem in amplifying, cloning, sequencing, maintaining in bacteria, and gene synthesis (12,19).

The k39 gene contains antigenic epitopes with amino acid variation affecting immunogenicity across *Leishmania* strains (20). The rk39 antigen that we used in the present study was prepared from an Iranian strain of *L. infantum* in the attempt to achieve the best diagnostic efficacy in Iran.

The findings of this study show that rK39-ELISA detected antibodies in symptomatic cases achieving a high sensitivity of 100% for human sera and 97.6 % for dog sera. Most studies so far have reported a high level of sensitivity for rK39 in the acute phase of the disease when high titers of antibodies are present in the serum (11,13,16,17,21,22). rK39-ELISA has shown 100% sensitivity in parasitologically confirmed CVL and high antibody-titer dogs in Venezuela, Turkey, Italy and Morocco (23–26). Hence, there is a significant statistical correlation between the antibody titer and sensitivity 27–29).

Furthermore, a meta-analysis of published data has shown a lower level of sensitivity for rK39 RDT in dog with clinical infection (87%) compared to symptomatic humans (94%) infected with *L. donovani* or *L. infantum* (30). Our data agree with published results. It is possible that this is due the different ability of humans and dogs to respond to a rK39 *immunodominant epitope*.

On the contrary, low sensitivity of rK39-ELISA (62.5%) has been observed in self– healing or sub-clinical human infection. Sensitivity ranging was described from 52.9% to 64.7% to diagnose infection in asymptomatic CVL (31). Moreover, similar results were reported in sera with low titers of antibodies by rK39 dipstick test in asymptomatic HVL (32,33). The antibody titer is directly related to the tissue parasitic load.

The sensitivity of CVL and HVL were 93.1% and 84.6%, respectively by CA-ELISA in cases with clinical signs, while a lower positivity of approximately 59.4% was seen in sera with lower levels of antibodies (≤1600(. Similarly, a low sensitivity (30%) was observed for CSA-ELISA in this group (31). The reasons for higher sensitivity of CA-ELISA in symptomatic dogs compared to the same status in humans are unknown; this finding might indicate that CA is more immunodominant than rK39 in dogs as compared to humans.

Interestingly, in spite of a remarkable surge of sensitivity (84.6%) for CA-ELISA in HVL with clinical manifestations compared to another group, it fails to reach a good agreement in the association with DAT (*k* =0.340). By contrast, this value is acceptable in dogs with a high titer of anti-body *(k* =0.763).

Our results indicate the lower specificity (86%) of ELISA using the rK39 in human in comparison with specificity reported from many VL-endemic countries, such as India and Nepal (97% to 98%) (13). This finding was similar to the study also reported the same specificity (85%) for diagnosis of HVL with rK39sub-produced from an Iranian strain of *L. infantum LON49*(6). This value was determined 94.4% when rK39-ELISA was used to detect antibody in the canines. A similar specificity has been observed in previous studies(30,34,35). However, cross-reactivity did not observe for rK39-ELISA in both hosts, the less specificity of rK39 in Iran in comparison with Europe is likely due to higher prevalence of other infectious diseases with similar signs.

The cross-reactivity of VL-negative sera infected by cutaneous leishmaniasis and tuberculosis were seen for CA, while rK39 protein did not react with anti-bodies of other infectious diseases despite the previous studies reported a cross-reactivity of rK39 RDT with CL caused by *L. tropica* in dogs (36,37). This finding indicated that the new antigen is more specific than the rK39 using in RDT for CVL.

In addition, the sensitivity of rK39 (100%) was found higher than rK39-sub (95%) prepared by Iranian researcher to detect antibody in people who have clinical signs (≥3200) (2). This finding shows different and higher density of immunodominant epitopes of rK39 causing more sensitivity and better assay reproducibility.

## Conclusion

New rk39 recombinant antigen from an Iranian strain of *L. infantum* seems to be used for diagnosis of VL in humans and dogs. Future attempts to design a double blind study to determine final validity of the antigen in the endemic areas of VL in Iran where *L. infantum* is the main causative agent for this disease.
